# Fucaceae: A Source of Bioactive Phlorotannins

**DOI:** 10.3390/ijms18061327

**Published:** 2017-06-21

**Authors:** Marcelo D. Catarino, Artur M. S. Silva, Susana M. Cardoso

**Affiliations:** Department of Chemistry & Organic Chemistry, Natural Products and Food Stuffs Research Unit (QOPNA), University of Aveiro, Aveiro 3810-193, Portugal; mcatarino@ua.pt (M.D.C.); artur.silva@ua.pt (A.M.S.S.)

**Keywords:** seaweeds, algae, Fucaceae, phlorotannins, bioactivities, antioxidant, antidiabetes, anti-inflammatory, antitumor, bioavailability

## Abstract

Fucaceae is the most dominant algae family along the intertidal areas of the Northern Hemisphere shorelines, being part of human customs for centuries with applications as a food source either for humans or animals, in agriculture and as remedies in folk medicine. These macroalgae are endowed with several phytochemicals of great industrial interest from which phlorotannins, a class of marine-exclusive polyphenols, have gathered much attention during the last few years due to their numerous possible therapeutic properties. These compounds are very abundant in brown seaweeds such as Fucaceae and have been demonstrated to possess numerous health-promoting properties, including antioxidant effects through scavenging of reactive oxygen species (ROS) or enhancement of intracellular antioxidant defenses, antidiabetic properties through their acarbose-like activity, stimulation of adipocytes glucose uptake and protection of β-pancreatic cells against high-glucose oxidative stress; anti-inflammatory effects through inhibition of several pro-inflammatory mediators; antitumor properties by activation of apoptosis on cancerous cells and metastasis inhibition, among others. These multiple health properties render phlorotannins great potential for application in numerous therapeutical approaches. This review addresses the major contribution of phlototannins for the biological effects that have been described for seaweeds from Fucaceae. In addition, the bioavailability of this group of phenolic compounds is discussed.

## 1. Introduction

**Figure 1:**
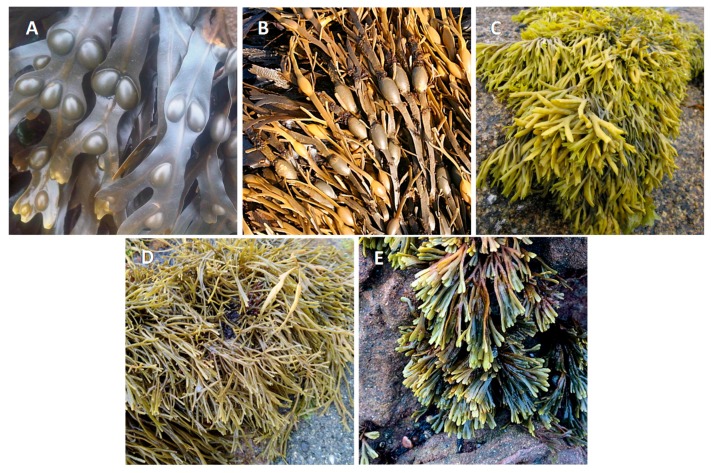
Type species of each genus composing the Fucaceae family. (**A**) *Fucus vesiculosus* L., photo by Emőke Dénes licenced by CC BY-SA/resized from the original; (**B**) *Ascophyllum nodosum* (L.) Le Jolis, photo by Anne Burgess licensed by CC BY-SA/resized from the original; (**C**) *Pelvetia canaliculata* (L.) Decaisne & Thuret, photo by Tom Corser licensed by CC BY-SA/resized from the original; (**D**) *Silvetia compressa* (J. Agardh) E. Serrão, T.O. Cho, S.M. Boo & Brawley, photo by Plocamium licensed by CC BY-NC/resized from the original; and (**E**) *Pelvetiopsis limitata* (Setchell) N.L. Gardner, photo by Peter D. Tillman licensed by CC BY/resized from the original.

Fucaceae is a family of brown algae containing five subordinate taxa currently recognized, including *Ascophyllum*, *Fucus*, *Pelvetia*, *Pelvetiopsis* and *Silvetia* (Figure 1), which dominate the biomass in the intertidal areas of many cold and warm temperate regions in the Northern Hemisphere, being distributed along the Northeast-Atlantic coastlines, from the White Sea to the south of the Canary Islands, and the Northwest-Atlantic, from south Greenland to North Carolina, as well as along the Northeast-Pacific coastline, extending from Alaska to California [1,2]. *Fucus* is undoubtedly the most prominent genus from this family. It currently comprises 66 taxonomically accepted species, which are characterized by a greenish brown trisected thallus, i.e., a structure consisting of a holdfast, a small stipe and flattened dichotomously-branched blades with terminal receptacles that swell during the reproductive season. The blades usually have a central-thickened area called the midrib, and in some species, such as *F. vesiculosus*, air bladders can be found to keep them floating in a vertical position when submerged [3]. This is also the most widely-distributed genus from Fucaceae being scattered throughout all of the regions covered by this family [4,5]. The most well-known species of this genus is *F. vesiculosus*, that commonly dominates the shallow macroalgae communities growing on high salinity waters from 0.5–4 m in depth and forming large belts that constitute the habitats for species-rich epiphytic and epibenthic communities [6,7].

*Ascophyllum* and *Pelvetia* are two monotypic genera, i.e., each comprises solely one species, namely *A. nodosum* and *P. canaliculata*, respectively, and are both exclusive to the North-Atlantic, although the latter is only found in the European coastlines [1,5,8,9,10]. As the most tolerant species to the exposure conditions, *P. canaliculata* forms a zone at the upper region of the shore, sometimes growing among coarse grass and other longshore angiosperms [11].

On the other hand, *Pelvetiopsis* and *Silvetia* are two genera from Fucaceae that are exclusive to the North-Pacific, the former distributed from south Canada to north California, while the latter covers the west coast of North America and has also been reported to occur in the Japan, China and Korea coastlines [10,12,13]. *Silvetia* species were originally classified as members of *Pelvetia*; however, owing to differences in oogonium structures and rDNA sequences, the new genus was created in 1999 [14]. Due to the lack of scientific interest in these two genera, little is known about them.

The use of Fucaceae, alongside other seaweeds, has long been part of human activities with applications in the most varied fields. Historically, *Ascophyllum*, *Fucus*, *Pelvetia* and *Silvetia* have been harvested and used as a food source for humans, typically in countries from Far East Asia, where seaweed consumption is part of their culture. Furthermore, although with less incidence, some *Fucus* species have also been consumed as foods in coastal countries of Western Europe and Alaska [15]. In the Azores Islands, the swollen receptacles of *F. spiralis* are a popular delicacy, known as sea lupines and eaten fresh [16].

Besides being used as food, *Ascophyllum* sp. and *Fucus* spp. have been used for distinct purposes over the centuries. Note that these seaweeds are often known as kelp, which is the name of the alkaline ashes produced from brown algae and used as an alkali agent for soap, paper and glass production, dying and in linen bleaching during the eighteenth–nineteenth centuries [17,18]. Later in the 1940s, *A. nodosum* was the most important feedstock for the business of alginate production in countries such as Ireland, Scotland and Norway, which were the principal suppliers of this phycocolloid [18,19]. However, because this species is relatively costly to harvest and it has a lower extract quality compared to other species, its use for this purpose has dramatically decreased during the recent years and been replaced by more attractive and versatile seaweeds including *Laminaria hyperborea* and *Lessonia* spp. [20]. Nevertheless, due to their combination of macro- and micro-nutrients, as well as the presence of natural plant growth hormones and other biostimulants, *A. nodosum* and, to a lesser extent, *Fucus* spp. continue to be used as biofertilizers [21,22,23,24], animal nutrition [25,26,27] and pest control [28,29,30,31]. Indeed, nowadays, *A. nodosum* has found its major application in the fertilizers, animal feed and phytopharmaceuticals industries, it being possible to find a series of *A. nodosum*-based products, such Acadian^®^, Agri-Gro Ultra, Alg-A-Mic, Maxicrop, Nitrozime, Soluble Seaweed Extract, Stimplex^®^, Tasco^®^ and several others currently available on the market.

In turn, the current most popular application of *Fucus* spp. is for the treatment of goiter, i.e., the swelling of thyroid, and thyroid-related complications caused by iodine deficits. In fact, *F. vesiculosus* along with *Laminaria* sp. were the original sources of iodine, found in 1811 by Bernard Courtois [32]. This element was further described by Moro and Basile [33] as the most important active principle of *F. vesiculosus*, since it is essential for the production of thyroid hormones, which in turn are responsible for the increase of the metabolism in most tissues and consequently raise the basal metabolic rate [32]. Because of that, *F. vesiculosus* supplements are commonly used not only for the treatment of goiter, but also for treating obesity [34]. *F. vesiculosus* has also been commonly used for the treatment of rheumatoid arthritis, asthma, atherosclerosis, psoriasis and skin diseases, as well as several other complications [35,36,37]. Likewise, *Ascophyllum* and *Pelvetia* are endowed with several medicinal properties including antioxidant [38,39], anticoagulant [40,41], anti-inflammatory [41,42], antitumor [43,44] and antidiabetic [45,46,47], among others. In addition, these Fucaceae can be currently found in the ingredient labels of a dozen cosmetic products used as antiaging, anti-wrinkle, anti-photoaging, slimming, moisturizing and skin-whitening agents [48,49].

**Figure 2:**
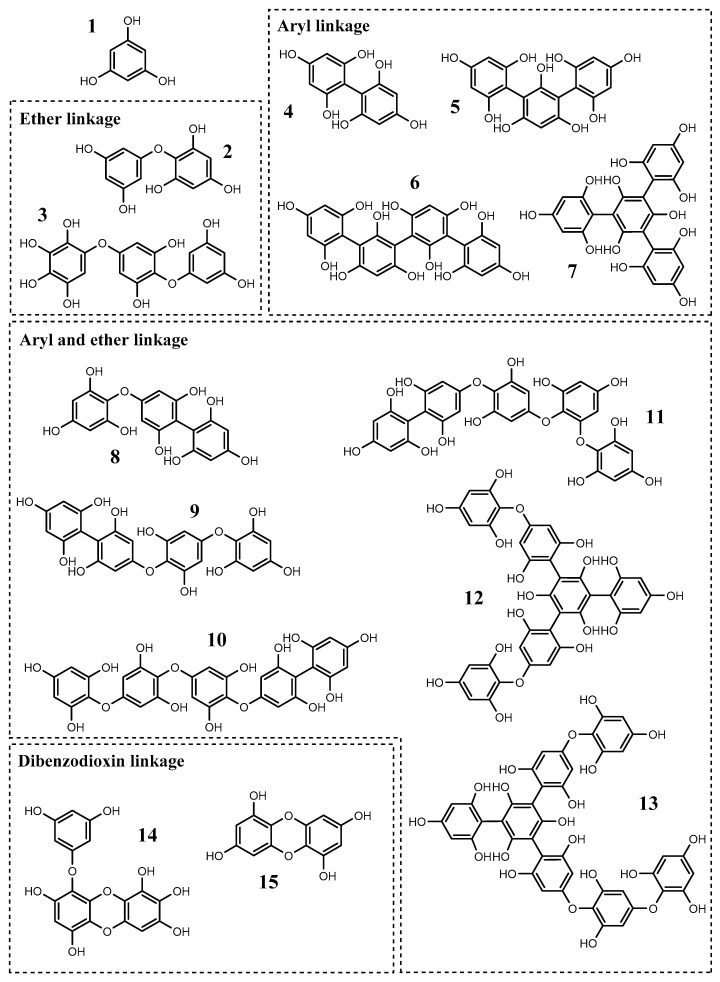
Structure of phlorotannins isolated from algae belonging to Fucaceae: (**1**) phloroglucinol; (**2**) diphlorethol; (**3**) hydroxytrifuhalol; (**4**) difucol; (**5**) trifucol; (**6**) tetrafucol A; (**7**) tetrafucol B; (**8**) fucophlorethol; (**9**) fucodiphlorethol; (**10**) fucotriphlorethol A; (**11**) fucotriphlorethol E; (**12**) trifucodiphlorethol A; (**13**) trifucotriphlorethol A; (**14**) 7-hydroxyeckol; and (**15**) phloroglucinol C–O–C dimer.

Among the various Fucaceae secondary metabolites, one can detach the importance of the phlorotannins, i.e., a class of phenolic compounds that is found exclusively in marine organisms, particularly in brown macroalgae [50]. These are very hydrophilic compounds, consisting of dehydro-oligomers or dehydro-polymers formed through C–C and/or C–O–C oxidative coupling of phloroglucinol (1,3,5-trihydroxybenzene) monomeric units, which are biosynthesized through the acetate–malonate pathway [51]. Phlorotannins may be found in a wide range of molecular sizes, comprised between 126 Da and 650 kDa [52], and according to the number of hydroxyl groups and nature of the structural linkages between phloroglucinol units, they can be characterized into four different subclasses: fuhalols and phlorethols (possessing an ether linkage), fucols (possessing an aryl linkage), fucophlorethols (possessing an ether and aryl linkage) and eckols and carmalols (possessing a dibenzodioxin linkage) [53]. From these subclasses, the most commonly found in Fucaceae are fucols and fucophlorethols (Figure 2).

Phlorotannins have been suggested to be multifunctional in brown seaweeds, with putative roles as primary cell wall components also involved in its biosynthesis and defensive mediators against natural enemies, working as herbivore deterrents, digestive inhibitors and as antibacterial and antifouling agents [54,55,56]. Besides, they also contribute to the protection of algae against ultraviolet radiation (UV-B) and may act as chelators of metal ions [57,58,59]. These compounds are known to accumulate mainly in the cell cytoplasm in specialized membrane-bound vesicles named physodes, representing up to 25% of seaweed´s dry weight (DW) [54].

During the last few years, an increasing interest has been paid to these algal metabolites since they have been demonstrated to exert numerous biological activities with potential application in food, pharmaceutical and cosmetic industries, among others. Because of their high abundance in phlorotannins, most studies involving the bioactivities of these phenolic compounds have been performed mainly with Laminariales, particularly those belonging to the Lessoniaceae family including *Ecklonia* spp. and *Eisenia* spp., while other algae families, such as Sargassaceae or Fucaceae, which could also represent a good source of these compounds, remain virtually unexploited. In this context, Fucaceae is of particular interest since, contrary to Sargassaceae (with some exceptions), most of the species from this family are considered edible. Therefore, and because of Fucaceae’s abundance in phlorotannins, these seaweeds might be of particular economic interest as they own great potential to be used as natural raw ingredients in foods, nutraceutical or pharmaceutical industries [60]. In this context, this manuscript revises the major biological properties described so far for the Fucaceae family, with special focus on their phlorotannin composition and importance for such effects, hoping to contribute to boost their industrial interest and utilization.

## 2. Phlorotannins from Fucaceae

Although some authors have reported the presence of phenolic acids and flavonoids in brown algae [61,62], phlorotannins represent their major phenolic constituents and, therefore, also in Fucaceae [63]. In fact, phlorotannins have been reported as the only phenolic compounds in *F. vesiculosus* [64,65]. According to what was revised by Holdt et al. [60], the highest phlorotannin contents registered for *A. nodosum* and *Fucus* sp. are 14% and 12% dry weight, respectively. Nevertheless, Fucaceae phlorotannins are very susceptible to inter-species variations. Connan et al. [66] observed that species such as *A. nodosum* and *F. vesiculosus* growing in the mid-tide zone have the highest content in phenolics (about 5.8% dry weight), while those growing in the lower intertidal level, such as *F. serratus*, have a lower phenolic content (4.3% dry weight), and the species growing at the upper level of the intertidal zone, such as *F. spiralis* and *P. canaliculata*, contain the lowest phenolic content (3.9% and 3.4% dry weight, respectively). Moreover, phlorotannins are also subject to significant intra-species variability depending on several factors such as algae size, age, tissue type and environmental factors, including nutrients, light, salinity, water depth and season [67]. The seasonal variations observed by Ragan and Jensen for *A. nodosum* and *F. vesiculosus* indicated that the polyphenols content was minimum (approximately 9–10% and 8–10% of dry matter, respectively) at the end of spring, during the period of fertility, and maximum (approximately 12–14% and 11–13% of dry matter, respectively) during the winter [68]. However, contradictory results were later reported, revealing that the phlorotannin peak of these Fucaceae occurs during the summer, matching with the higher solar exposure period and thus agreeing with the UV-protective functions invoked for these compounds [66]. Likewise, in Pavia and Toth’s [67] experiments, the authors observed that the thalli from *A. nodosum* and *F. vesiculosus* that had been exposed to sunlight contained higher phlorotannins than the shaded ones. Observations of maximum phlorotannin content in the summer, when the irradiance is highest, has been described for other Fucales as well [69,70,71], and current evidence suggests that the production of phlorotannins by seaweeds is tightly correlated with UV radiation [72,73,74].

Salinity is another parameter considered determinant for phlorotannin concentrations in seaweeds since, according to Pedersen [75], the phenolic content of *A. nodosum* and *F. vesiculosus* increases with increasing salinity in their habitats. Further research confirmed that the decrease of the salinity coincided with high exudation of *A. nodosum* and *F. vesiculosus* phenolics into the surrounding water, thus resulting in a significant reduction of the phenolic content of these two species [58].

Identification and characterization of phlorotannins from brown algae has been a challenging subject since, in addition to their high susceptibility to oxidation and lack of commercially available standards, the large size and complexity, structural similarity and reactivity with other compounds make them very difficult to isolate and purify from such polymeric mixtures as crude seaweed extracts [76,77]. Therefore, the exact characterization of phlorotannins commonly requires the combination of ultra-performance liquid chromatography (UPLC) (equipped with column technologies capable of resolving extremely polar complex polymer mixtures) with mass spectrometry (MS) and nuclear magnetic resonance (NMR) techniques [78,79]. Nevertheless, a few works have already focused on the phlorotannin profile from *Fucus* spp., *Pelvetia canaliculata* and *Ascophyllum nodosum* in terms of degree of polymerization (DP). In *A. nodosum* and *P. canaliculata*, phlorotannins of DP 6–13 were found to be predominant, while *F. spiralis* was particularly rich in compounds with lower DP (4–6) [79]. Similarly, Steevensz et al. [80] reported that higher DP phlorotannins were observed with more abundance in *P. canaliculata* > *F. vesiculosus* > *A. nodosum > F. spiralis*. Interestingly, phloroglucinol monomers up to 39 units were detected in all of these seaweeds, except *P. canaliculata*, which contained phlorotannins composing up to 49 monomeric units. This fact has been hypothesized by the authors to be correlated with the higher exposure of this species to extreme conditions, consequently requiring more complex phlorotannin structures for their protection.

In addition to DP studies, some authors were also able to isolate and identify phlorotannins from *F. vesiculosus* including phloroglucinol (1 in Figure 2), difucol, trifucol and tetrafucols A and B; fucophlorethol, fucodiphlorethol and fucotriphlorethols A and E; trifucodiphlorethol A and trifucotriphlorethol A (4–13, respectively, in Figure 2) [77,81,82,83,84]. More recently, compounds such as hydroxyfuhalol A, difucol/diphlorethol, tetrafucol, fucodiphlorethol, 7-hydroxyeckol and the C–O–C dimer of phloroglucinol (2–4, 6, 9, 14 and 15, respectively, in Figure 2) were identified in *A. nodosum* extracts, as well [85].

## 3. Biological Activities

Although phlorotannins have been subject to thorough research focusing on their numerous potential biological activities, the majority of these studies have been performed with extracts from *Ecklonia* spp. or *Eisenia bicyclis* [52]. Still, interesting results focusing on phlorotannins extracted from brown algae belonging to Fucaceae, mostly from *Fucus* spp. and *Ascophyllum nodosum*, have arisen. Phenolic extracts from these algae have been demonstrated to exhibit various biological activities including antioxidant, anti-inflammatory, antimicrobial, antidiabetic and several others that could be of great interest for the development of new functional and/or therapeutic agents with high value for the food and pharmaceutical industries, thus strengthening the commercial exploitation of such macroalgae.

### 3.1. Antioxidant Activity

As phenolic compounds, the most characteristic biological effect of phlorotannins is the antioxidant activity. Among four species of brown algae, including *Cystoseira nodicaulis*, *Himanthalia elongata*, *F. serratus* and *F. vesiculosus*, the latter exhibited a total phenolic content (TPC) of 232.0 μg phloroglucinol equivalents (PE)/mg ethanolic extract, corresponding to the extract with the highest phenolic abundance, and the strongest activity in ferric reducing antioxidant power (FRAP) and 1,1-diphenyl-2-picrylhydrazyl radical (DPPH^●^) assays (307.3 μg trolox equivalents (TE)/mg extract and IC_50_ = 4 μg/mL, respectively). In turn, *C. nodicaulis* ethanolic extract, which yields the lowest phenolic content (89.1 μg PE/mg extract), tendentially revealed the lowest antioxidant activity in these two methods (101.4 μg TE/mg extract and IC_50_ = 28.0 μg/mL, respectively) [86]. A similar study performed with ten species belonging either to green, red or brown algae revealed that the group of Fucaceae (*F. vesiculosus*, *F. serratus* and *A. nodosum*) gave origin to the richest acetone (70%, *v*:*v*) extracts in terms of phenolic content, representing 24.2, 24.0 and 15.9 g PE/100 g extract, respectively. Likewise, these three were the most active antioxidant extracts, revealing EC_50_ values of 10.7, 11.0 and 18.5 μg/mL, respectively, in DPPH^●^ and oxygen radical antioxidant capacity (ORAC) values of 2.57, 2.55 and 1.42, respectively, against the >25.8 μg/mL and >0.98 mmol TE/g extract observed for the remaining extracts [87]. Nevertheless, although this evidence indicates a strong correlation between antioxidant activity and total phenolic content, this might not always be true in every case. In fact, according to O’Sullivan and co-workers [88], despite that the total phenolic content of the methanolic extract (60%, *v*:*v*) from *F. vesiculosus* only accounted for 2.5 mg gallic acid equivalents (GAE)/g DW, this exhibited an overall antioxidant activity in the FRAP (109.8 μM ascorbic acid/g DW), DPPH^●^ (31.2% radical scavenging) and β-carotene bleaching inhibition (71.2% protection) assays, which was better than the equivalent extracts from *A. nodosum* (81.0 μM ascorbic acid/g DW, 25.6 and 76.3%, respectively), *P. canaliculata* (71.5 μM ascorbic acid/g DW, 7.3 and 53.9%, respectively) and *F. serratus* (113.5 μM ascorbic acid/g DW, 5.5 and 62.2%, respectively), all containing approximately 4 mg GAE/g DW. Although this fact could result from the contribution of non-phenolic compounds present in the extract, it may also suggest that more important than the total phenolics content in the extract is the nature of such compounds. Indeed, when considering the specific activity of phlorotannins, Breton et al. [89] observed that the oligophenols fraction (<2 kDa) from *A. nodosum* methanol 100% extract revealed an antioxidant index (AI_50_) values (i.e., the amount of phenols in μg contained in the fraction necessary to obtain 50% of inhibition in the DPPH^●^ assay) below 20 μg, whereas the fraction of >50 kDa phenols exhibited AI_50_ values of 34 μg, thus evidencing the importance of the molecular weight for the physiological roles and putative function of phlorotannins. Through an electrochemical approach, the specific activity was also found stronger for subfractions of 2–50 kDa and <2 kDa isolated from *A. nodosum* methanol 100% extracts, rather than subfractions over 50 kDa (AI_50_ = 0.24, 0.78 and 1.24 × 10^3^ μM PE/L, respectively), and 1–4-times more active than the corresponding subfractions obtained from the crude methanol 50% extract, indicating differences on phlorotannins activity based on polarity [90]. However, when testing the relationships between the degree of polymerization, molecular size and antioxidant activity of different molecular weight subfractions obtained from *F. vesiculosus* ethanol 80% extracts, no clear correlations were found, except for the Fe^2+^ chelating ability, which was greater for the 100–300 and >300 kDa subfractions (47.6 and 45.1%, respectively) than for the 30–100, 5–30 and <5 kDa subfractions (36.6, 33.7 and 25.1%, respectively) [91].

Cérantola et al. [92] showed that fucol and fucophlorethol polymers, both isolated from *F. spiralis*, presented identical Q_50_ values (approximately 33 μg), i.e., the amount of compound in μg necessary to obtain 50% of inhibition in DPPH^●^ assay, which in turn were lower than those obtained for ascorbic acid and phloroglucinol (38.2 and 41.7 μg, respectively), thus evidencing higher antioxidant activity than these two reference compounds. More recently, positive results were observed for three phlorotannins isolated from *F. vesiculosus*, namely trifucodiphlorethol A, trifucotriphlorethol A and fucotriphlorethol A, which revealed good DPPH^●^ scavenging activity (IC_50_ = 14.4, 13.8 and 10.0 μg/mL, respectively) comparable to that of phloroglucinol (IC_50_ = 13.2 μg/mL), as well as a potential for scavenging peroxyl radical three-times more strongly than that of trolox in the ORAC assay. Additionally, moderate inhibitory effects towards xanthine oxidase activity were observed for trifucotriphlorethol A [84].

Because of these promising antioxidant effects, Fucaceae seaweeds are endowed with a great potential for the development of novel antioxidant products with high commercial interest for pharmaceutical, nutraceutical, cosmetic and especially food industries. Indeed, the introduction of Fucaceae phlorotannin extracts in food matrixes has already been demonstrated to effectively act as rancidification inhibitors/retarders, thus contributing for the enhancement of their shelf-lives and standing out as good candidates for exploitation as natural food additives. In this context, Honold et al. [93] found that introducing 1.5–2 g/kg of *F. vesiculosus* ethanol 80% or acetone 70% extracts in fish-oil-mayonnaise resulted in a significant enhancement of the product’s oxidative stability by reducing the hydroperoxides’ formation and lipid oxidation reactions. In a similar study conducted with fish muscle, the addition of 300 mg/kg muscle of oligomeric purified phlorotannin subfractions from *F. vesiculosus* was capable of inhibiting the lipid peroxidation of the product, demonstrating an effectiveness comparable to that of 100 mg/kg propyl gallate, one of the most potent antioxidant additives in food systems [94]. O’Sullivan et al. [95] also observed that the introduction of 0.5% (*w*/*w*) of *F. vesiculosus* ethanolic extracts into raw milk had promising effects against lipid oxidation of this dairy product, although it was not well accepted from a sensorial perspective due to the green/yellowish color and fishy taste. Further studies conducted by the same research group showed that the incorporation of 0.5% (*w*/*w*) *A. nodosum* or *F. vesiculosus* extracts (ethanolic 80% and 60%, respectively) into yogurts resulted in good inhibitory effects against lipid oxidation, without affecting the product’s acidity, microbiology or whey separation parameters. Once again, introduction of *F. vesiculosus* extract was sensorially rejected, while yogurts with *A. nodosum* extract were generally well accepted by the panelists [96]. The promising antioxidant effects of Fucaceae phenolic extracts have also been demonstrated in cellular models (Table 1).

In Wang et al. work [91], five phlorotannin subfractions from *F. vesiculosus* (separated by dialysis according to their different molecular weights) produced a decrease in ROS production inversely proportional to the compounds molecular weight in phorbol-12-myristate-13-acetate (PMA)-induced human mononuclear cell primary cultures. Indeed, incubation of Raw 264.7 macrophages with two *F. vesiculosus* ethanolic extracts (Ext1, 35%, and Ext2, 70%) resulted in the reduction of PMA or lipopolysaccharide (LPS)-stimulated O_2_^●−^ production, the former showing IC_50_ values of approximately 38 μg/mL in both assays, while the latter was more effective towards PMA rather than LPS stimulation (IC_50_ = 31 and 68 μg/mL, respectively) [97]. *A. nodosum* phlorotannin extract at 0.2% was also shown to significantly reduce the *tert*-butyl hydroperoxide (*t*-BHP)-induced ROS production in epithelial cells to levels close to the negative control [38].

Quéguineur et al. [99] further observed that a digested-dialyzed phlorotannin extract (rich in compounds over 1 kDa) from *A. nodosum* not only caused the reduction of intracellular ROS and lipid peroxidation in *t*-BHP-induced HepG-2 cells, as also enhanced their endogenous antioxidant defenses by increasing the levels of glutathione (GSH) and the enzyme activities of GSH-peroxidase (GSH-px), GSH-reductase (GSH-red) and GSH-S-transferase (GSH-tr). Augmented levels of GSH (32–39% higher than the control) were observed as well on Caco-2 cells incubated not only in the presence of *A. nodosum* hydromethanolic extract at 100 μg/mL, but also with those of *P. canaliculata*, *F. vesiculosus* and *F. serratus*. Furthermore, all of these extracts, particularly that of *P. canaliculata*, could almost completely restore the H_2_O_2_-induced depletion of superoxide dismutase (SOD) activity, although only two Fucaceae extracts, namely *F. serratus* followed by *F. vesiculosus*, exhibited a reduction of H_2_O_2_-induced oxidative damage to DNA (from 63% in control to 53% and 50%, respectively) [88]. The same *F. serratus* and *F. vesiculosus* methanolic 50% extracts were posteriorly confirmed to exhibit DNA protective effects in Caco-2 cells treated with H_2_O_2_, but not with *t*-BHP, although both *F. serratus* alongside with *P. canaliculata* extracts completely restored the SOD activity that was impaired by the *t*-BHP stimulation [39]. However, when testing extracts obtained by different procedures, instead of observing DNA protective effect against H_2_O_2_-induced oxidative damage, *F. serratus* (aqueous and ethanolic 80% extracts) and *F. vesiculosus* (methanolic 60% extract) revealed a decrease of the *t*-BHP-induced DNA damage of approximately 50% compared to the non-treated Caco-2 cells, most likely due to the extraction of phlorotannins with different polarities. In turn, *A. nodosum* ethanolic 80% extracts exhibited DNA protective effects either in the presence of H_2_O_2_ or *t*-BHP, while the ethanol 60% extract was only active against H_2_O_2_ and the aqueous extract was effective against *t*-BHP [98]. Hence, overall, the above-mentioned works point out the promising effects of phlorotannins of Fucaceae origin towards distinct oxidative stress events. Still, it is relevant to note a common flaw in all of these studies, i.e., the lack of comparison of the antioxidant activities of these seaweed extracts and/or phlorotannin compounds with that of well-known compounds. The gathering of this information would be helpful to achieve a better comprehension of the actual potential of these compounds.

In vivo experiments conducted by Zaragozá et al. [97] revealed that the feeding of Sprague–Dawley rats with *F. vesiculosus* phenolic extracts resulted in an increased blood plasma antioxidant activity slightly better than that of phloroglucinol, which is commonly used as a standard compound. In more detail, after a four-week oral treatment of 200 mg/kg body weight/day of *F. vesiculosus* ethanol 70% extract, the reducing power, paraoxonase 1 (PON-1) activity and O_2_^•−^ scavenging activity in the plasma were increased by 29%, 33% and 25%, respectively. Phloroglucinol administered in the same conditions also produced positive, although slightly lower effects in these parameters, causing a 31% and 12% increase of reducing power and PON-1 activity, respectively, and no activity against O_2_^•−^. The fact that thiobarbituric acid reactive substances (TBARS) were also reduced by 17% in the *F. vesiculosus-*treated rats and 12% in phloroglucinol-treated group might be a consequence not only the plasma’s increased ability to scavenge free radicals, but also PON-1’s greater hydrolytic activity. Particularly, in the phloroglucinol-treated group, in which no effects were seen on O_2_^•−^, the increase of this enzyme activity might be the major cause for TBARS reduction since this enzyme is known for protecting low-density lipoproteins from oxidative modification by ROS and contributing for the degradation of hydrogen peroxide (peroxidase activity) [100].

In several studies, phloroglucinol was proven to display a very pleiotropic role in oxidative stress events. Indeed, this compound was shown to reduce several oxidative stress hallmarks in numerous cell lines, stimulate the intracellular antioxidant defenses including the activation of nuclear factor (erythroid-derived 2)-like 2 (Nrf2) [101,102,103,104,105,106] and even positively contribute for photoprotective effects on skin [107] and improvement of motor functions and oxidative damage in the brain of animal models of Parkinson’s disease [105,108].

### 3.2. Antidiabetic Activity

In 2012, diabetes mellitus was the direct cause of 1.5 million deaths, reaching an estimated prevalence of approximately 9% among the worldwide adult population in 2014. Moreover, in 2030, it is projected that this disease will be the 7th main cause of death in the world [109]. In the specific case of type 2 diabetes mellitus, the most common therapeutic targets are α-amylase and α-glucosidase, two enzymes responsible for the starch hydrolysis releasing the glucose monomers for subsequent absorption by the small intestine. Therefore, the inhibition of these enzymes reduces the availability of free glucose monomers and consequently decreases the postprandial peak of blood glucose levels [110].

In this context, phenolic extracts from *Fucus* spp. and particularly from *A. nodosum* have demonstrated promising effects against these enzymes (Table 2). Per Zhang et al. [45], the inhibitory effects of different fractions from *A. nodosum* ethanol 50% extracts towards α-glucosidase activity was highly correlated with their phlorotannin content, as the lowest IC_50_ value (24.0 μg/mL) was observed for the C18 purified ethyl acetate fraction (TPC = 70.2% PE), followed by non-purified ethyl acetate fraction (IC_50_ = 38.0 μg/mL; TPC = 39.8% PE) and crude ethanol extract (IC_50_ = 77.0 μg/mL; TPC = 22.5% PE). When comparing the α-glucosidase inhibitory activities of ethanol 96% and acetone 70% extracts from *A. nodosum* and *F. vesiculosus*, both rich in phlorotannins, to that of acarbose (i.e., a well-known inhibitor of α-glucosidase and α-amylase currently used as an antidiabetic drug), IC_50_ values of 8.9 and 0.72 μg/mL, respectively, for the former, and 4.4 and 0.34 μg/mL, respectively, for the latter were obtained, corresponding to an inhibitory activity 160–2000-times stronger than that of acarbose (IC_50_ = 720 μg/mL) [111]. The methanolic extract of *P. siliquosa* (currently *S. siliquosa*), also rich in phlorotannins, was shown to be an effective inhibitor of α-glucosidase, as well [112]. It should be noted that the biological effects of Fucaceae algae towards this enzyme can be significantly affected depending on the harvesting season. In the specific case of *A. nodosum*, the highest inhibitory activity against α-glucosidase was observed during the summer, more precisely in July, when the authors found the highest phlorotannin accumulation for this species [113].

In addition to the strong inhibitory effect against α-glucosidase, *A. nodosum* extracts were also proven to display inhibition towards α-amylase [46]. Indeed, an acetonitrile 50% extract from *A. nodosum* purified in a solid-phase extraction (SPE) column was shown to exert higher inhibitory activity on α-amylase rather than on α-glucosidase, with an IC_50_ value eight-times lower than that of acarbose (0.8 μg/mL) [114]. Similar results were described for phlorotannin-purified fractions of an extract from *F. distichus*, which were capable of reducing the activity of both α-glucosidase and α-amylase 126- and 10-times more effectively than the above-mentioned pharmaceutical drug [115]. The aqueous and ethanolic 80% extracts of *A. nodosum*, *F. vesiculosus*, *F. serratus*, *F. spiralis* and *P. canaliculata* presented inhibitory properties against these two enzymes comparable to that of acarbose as well, although depending on the extract procedure, some differences could be observed. In particular, for the aqueous extracts, the strongest α-amylase inhibitor was *A. nodosum* (IC_50_ = 53.6 μg/mL), followed by *F. vesiculosus* > *P. canaliculata* > *F. serratus* > *F. spiralis*, while for the ethanol extracts, *A. nodosum* (IC_50_ = 44.7 μg/mL) still exhibited the best activity, but the *P. canaliculata* extract was more active than that of *F. vesiculosus*. These differences were more evident in the case of α-glucosidase. The aqueous extracts from *F. vesiculosus* and *P. canaliculata* exhibited similar inhibitory activity (IC_50_ approximately 0.3 μg/mL), followed by *A. nodosum > F. serratus* > *F. spiralis*, while for the ethanol extract, *F. vesiculosus* maintained the strongest activity (IC_50_ = 0.49 μg/mL), but *P. canaliculata* activity was only followed by that of *F. spiralis*. It is worth noting that overall, the ethanol extracts were more effective against α-amylase, while the opposite was observable for α-glucosidase. Nevertheless, with the exception of *F. spiralis*, α-amylase inhibitory profiles of all aqueous and ethanolic extracts were very similar to that of acarbose. Notably, both *F. vesiculosus* extracts exhibited stronger α-glucosidase inhibitory activity than that of the pharmaceutical drug [47].

In Roy et al. [119], the incubation of α-amylase and α-glucosidase with a commercial mixture of *A. nodosum* and *F. vesiculosus* phlorotannin extract resulted in inhibitions of approximately 100% at concentrations below 0.2 μM. Furthermore, the immediate postprandial blood glucose levels of Wistar rats orally treated with this mixture (7.5 mg/kg) were decreased by 90%, and the peak increase of insulin secretion was reduced by 22%. In addition, in a previous study, the oral administration of 200 mg/kg/day of two different *A. nodosum* phlorotannin extracts (crude ethanol 50% extract and a HP-20 column purified ethanol 50% extract) to streptozotocin-diabetic mice fed with sucrose during four weeks was shown to improve the fasting serum glucose levels and lower the postprandial blood glucose level at the 14th day by 27% and 25% comparing to the diabetic controls [45]. Identical results were described for *P. siliquosa* (currently *S. siliquosa*) methanolic 70% extracts, which not only suppressed the enzymatic activities of sucrase and maltase in vitro (IC_50_ = 2.24 and 2.84 mg/mL), but also reduced the postprandial blood glucose levels in vivo on sucrose-fed Wistar rats orally treated with 1 g/kg body weight of this extract [112].

All these evidences suggest that the extracts from *A. nodosum* and/or *Fucus* spp. have great potential to be used either as an anti-diabetic therapeutic approach targeting α-glucosidase and α-amylase and/or as a co-ingredient of already existent pharmaceutical drugs. Indeed, Pantidos et al. [118] demonstrated that the combination of acarbose with a purified phlorotannin-rich fraction from *A. nodosum* exerted a synergistic inhibitory effect towards these two enzymes, thus allowing one to reduce the concentration of acarbose necessary for obtaining an effective inhibitory activity from 1.0–0.5 μg/mL. Moreover, in a human clinical trial, the single ingestion of 500 mg of a commercial extract mixture from *A. nodosum* and *F. vesiculosus* 30 min prior to the consumption of 50 g of carbohydrates was associated with a 12.1% reduction in the insulin incremental area of the curve and a 7.9% increase in insulin sensitivity [120].

Although the Fucaceae phenolic extracts have been mainly described for their acarbose-like effects when evaluating their anti-diabetic effects, other possible mechanisms were also reported. For example, the phenolic-rich ethanol extract from *A. nodosum* was shown to stimulate the basal glucose uptake into 3T3-L1 adipocytes, thus contributing to the reduction of blood glucose levels and the amelioration of hyperglycemia [45]. Furthermore, phloroglucinol alongside with four purified phlorotannin (from *F. vesiculosus* acetone 70% extract) fractions demonstrated very effective inhibitory activities against the bovine serum albumin (BSA)-methylglyoxal assay (IC_50_ = 58 μg/mL for phloroglucinol and approximately 160 μg/mL for algal fractions) and the BSA-glucose assay (IC_50_ = 68 μg/mL for phloroglucinol and 45–1526 μg/mL for algal fractions) and, therefore, a promising anti-advanced glycated end-products (AGEs) formation activity, i.e., a class of compounds generated by the exposure of proteins and other endogenous molecules to reducing sugars [116]. Due to the high blood glucose levels on diabetic patients, AGEs are produced in concentrations beyond the normal levels, thus leading to pathological consequences that are on the basis of the diabetic complications like retinopathy, nephropathy, neuropathy and cardiomyopathy [121]. Therefore, the ability of phloroglucinol and *F. vesiculosus* phlorotannins to prevent their formation indicate that they may contribute to the protection against the diabetic-related pathologies. Other studies with phloroglucinol demonstrated that it has the capacity to protect pancreatic β-cells from high glucose-induced oxidative stress and consequent apoptosis [122].

### 3.3. Anti-Inflammatory Activity

In addition to the previously mentioned biological activities, phlorotannins have also been closely related to the targeting of numerous inflammatory events. Note that inflammation is a complex and coordinated immunological response of the organism to harmful stimuli, consisting of a tightly regulated signaling cascade that is orchestrated by a series of pro-inflammatory mediators including cytokines, chemokines, adhesion molecules, enzymes and others [123]. Among these mediators, one can highlight the importance of tumor necrosis factor-α (TNF-α), whose main function is the activation of nuclear factor-κB [124], which in turn is responsible for the transcription of several genes encoding other pro-inflammatory mediators including TNF-α itself, interleukins (ILs), chemokines, adhesion molecules and key inflammatory enzymes including cyclooxygenase-2 and inducible nitric oxide synthase (COX-2 and iNOS, respectively), which further disseminate the pro-inflammatory stimuli [125]. Therefore, the described anti-inflammatory activities of Fucaceae phlorotannins are based on the screening of their ability to target one or multiple of these mediators (Table 3).

According to Zaragozá et al. [97], the production of NO^●^ (i.e., a pivotal free radical involved in the signaling and pathogenesis of inflammation) in PMA-stimulated RAW 264.7 cells was inhibited in a dose-dependent fashion by a phlorotannin-rich *F. vesiculosus* ethanol 35% extract (IC_50_ of 37 μg/mL). Likewise, a phlorotannin extract from *A. nodosum* was shown to dose-dependently decrease the LPS-induced expression of TNF-α and IL-6 in U937 macrophages [38]. Similar results were later observed in an identical cellular model, thus endorsing the hypothesis that phenolic extracts of this species could act as anti-inflammatory agents by blocking the propagation of the pro-inflammatory stimuli [126]. Bahar and co-workers [42] reported that the treatment of porcine colonic tissues ex vivo either with *A. nodosum* ethanol 80% or *F. serratus* aqueous extracts, caused a significant downregulation of the LPS-induced pro-inflammatory genes including *IL6*, *IL8* and *TNFA* (encoding for the cytokines IL-6, IL-8 and TNF-α, respectively), comparable to that of dexamethasone (i.e., a corticosteroid medication used for the treatment of inflammation and autoimmune diseases). More recently, this research group also observed that the treatment of TNF-α-challenged Caco-2 cells with an ethanol 80% extract of *A. nodosum* significantly suppressed the expression of several pro-inflammatory genes encoding cytokines (*IL8*, *TNFA*, *IL1B*, *IL18* and *CSF1*), chemokines (*CXCL10*, *CCL5*), components of the NF-κB pathway (*NFKB2* and *IKBKB*) and other mediators (*PTGS2* and *MIF*) by more than two-fold compared to the negative control. Further experiments in LPS-stimulated porcine colonic tissue ex vivo revealed that this *A. nodosum* extract caused the downregulation of immune-related genes, including *LYZ*, *IL8*, *PTGS2*, *TLR6*, *CXCL10*, *IL6*, *CXCL11*, *ICAM*, *NFKB1* and *CXCL2* [127]. Identical results were also reported for a cold water extract of *F. vesiculosus*, which inhibited the expressions (>2-fold) of the genes *IL17A* and *IL8* (encoding for cytokines), *CCL2*, *CXCL2*, *CXCL10* and *CXCL11* (encoding for chemokines), *ICAM1* and *VCAM1* (encoding for cell adhesion molecules), *TLR4* and *TLR7* (encoding for Toll-like receptors), *NFKB1* and *RELB* (encoding for NF-κB components), *MAP3K8* and *CJUN* (encoding for mitogen activated protein kinases and activator protein-1 components, respectively) and *PTGS2*, *C5* and *LYZ* (encoding for other pro-inflammatory mediators), in the same ex vivo model. Notably, Toll-like receptor 4 (TLR-4) was identified in this study as an important target for the anti-inflammatory effect of this extract [128]. It is interesting to note that dexamethasone (as shown in pig or in rat colonic tissue models) does not seem to interfere with the expression of TLR-4 [129,130]. However, further investigations are still needed in order to understand whether the *A. nodosum* anti-inflammatory bioactivity mediated through inhibition of TLR-4 expression has any distinct advantage over the inflammatory immune diseases treatments based on dexamethasone.

Such a broad spectrum of anti-inflammatory bioactivity of *A. nodosum* and *F. vesiculosus* suggests that there is a great potential for future exploitation of these seaweeds as therapeutic agents for the treatment of inflammatory conditions, particularly those related with mammalian intestine diseases, although further studies, namely in vivo, would be necessary to better evaluate the feasibility of these results.

*F. distichus* is another example of a Fucaceae with promising anti-inflammatory properties, comparable to those of dexamethasone. Kellogg et al. [131] reported that the fucophlorethols-rich fraction isolated from a methanolic 80% extract of this seaweed was remarkably effective against the expression of an array of inflammatory markers triggered by LPS-stimulation of RAW 264.7 macrophages, showing particular high activity towards COX-2, iNOS, IL-1β, IL-6, TNF-α, intercellular adhesion molecule-1 and TLR-4, reducing their expression to below 10% at 50 μg/mL when comparing to the LPS control. Monocyte chemoattractant protein-1, IL-17 and TLR-9 were also found strongly inhibited, below 60%, for the same concentration. Based on this data and on the fact that fucophlorethols are one of the most abundant phlorotannin groups in Fucaceae, it is possible to suggest that these compounds might be important contributors for the anti-inflammatory activity that has been observed for the phenolic extracts from this family.

### 3.4. Antitumor Activity

Both oxidative stress and inflammation have long been associated with the development of cancer. The production of ROS, including hydroxyl radical (OH^●^) and superoxide (O₂^●−^), and reactive nitrogen species (RNS), such as nitric oxide (NO^●^) and peroxynitrite (ONOO-), associated with chronic inflammatory states may lead to environments that foster genomic lesions and tumor initiation [132]. In this field, reported data suggest that Fucaceae phlorotannins can exert important chemopreventive and antiproliferative effects against some cancer cell lines (Table 4).

According to Nwosu et al. [114], a purified phlorotannin extract from *A. nodosum* origin was shown to strongly inhibit the proliferation of colon cancer cells in a dose-responsive manner, with IC_50_ values of 33 μg/mL. Likewise, an HPLC fraction obtained from an *F. vesiculosus* acetone extract was reported to have potent anti-proliferative effects on different pancreatic cancer cell lines, showing EC_50_ values between 17.4 and 28.9 μg/mL. The authors also mentioned that this extract affected only proliferating, but not resting cells through stimulation of cell cycle arrest, which is comparable to the effects of common chemotherapeutic drugs clinically used, such as gemcitabine [133]. Further studies from this research group concluded that the multistep fractionation of *F. vesiculosus* acetone extract through precipitation, normal phase HPLC and reversed phase HPLC could result in the obtainment of two active fractions (F15/16 and F36/37) against human pancreatic cancer (Panc89) (EC_50_ of approximately 16 and 47 μg/mL for F15/16 and F36/37, respectively) and human pancreatic cancer PancTu1 (EC_50_ of approximately 17 and 80 μg/mL for F15/16 and F36/37, respectively), despite that their anti-proliferative effects were far from those of the chemotherapeutic gemcitabine (EC_50_ = 3.5 ng/mL and 14 ng/mL against Panc89 and PancTu1 cells, respectively) commonly used as a first line treatment for pancreatic cancer [134]. Antitumor activity against HeLa cells was reported for an *F. spiralis* dichloromethane extract, which reduced their proliferation by 50% at 10.7 μg/mL. However, the phenolic content of this extract was only 13 μg GAE/mg extract, which makes phlorotannins unlikely to contribute for these results [135]. Nevertheless, a phlorotannin extract from this species, particularly abundant in fucophlorethols, was shown to inhibit the activity of hyaluronidase, an enzyme overexpressed in breast cancer, revealing an IC_50_ of 0.73 mg/mL DW, which was 2–4-times lower than the results observed for three other Fucales, namely *Cystoseira nodicaulis*, *C. usneoides* and *C. tamariscifolia* [136]. Still, one should note that these inhibitory effects are considerably lower when comparing with the IC_50_ reported for other compounds such as catechin (0.18 mg/mL), epigallocatechin gallate (0.09 mg/mL) or sodium cromoglycate (0.14 mg/mL), known as good inhibitors of this enzyme [137].

Focusing three fucophlorethols isolated from *F. vesiculosus*, namely trifucodiphlorethol A, trifucotriphlorethol A and fucotriphlorethol A, Parys et al. [84] reported that the good chemopreventive properties of these compounds were due to their capacity to inhibit the activity of aromatase (an enzyme also involved in the carcinogenesis from breast and other estrogen-related cancers) and CYP1A, which is an enzyme belonging to the cytochrome P450 family and known to be involved in carcinogen activation of mutagens derived from cooked food.

These data suggest that *A. nodosum* and *Fucus* spp. phenolic compounds could represent possible new agents with therapeutic applications on the treatment of pancreatic and colon cancer, the former being one of the most aggressive cancer entities and the latter one of the most incident cancers worldwide [138]. Still, much work needs to be carried out in order to prove both the efficacy and safety of these agents in vivo*.*

### 3.5. Other Biological Activities

The typical phlorotannin profile from brown algal with antimicrobial activity mainly consists of phloroglucinol, eckol and dieckol [139,140]. Fucaceae seaweeds are, however, more prevalent in fucols and fucophlorethols. Yet, some positive results in this field have already been reported. Indeed, Sandsdalen et al. [141] have shown that a fucophlorethol derivative isolated from *F. vesiculosus* was a potent bactericidal agent against both Gram-positive (*Staphylococcus aureus*, *Staphylococcus epidermidis*) and Gram-negative (*Escherichia coli*, *Proteus mirabilis*, *Pseudomonas aeruginosa*) bacteria, reducing their growth by 85% compared to the controls. Likewise, the phlorotannins purified from *F. spiralis* acetone 70% extract showed antibacterial effects against Gram-positive bacteria, exhibiting minimum inhibitory concentrations of 2 mg/mL for *Micrococcus luteus*, 2 mg/mL for *S. epidermidis*, 7.8 mg/mL for *S. aureus* and *Bacillus cereus* and 15.6 mg/mL for *Enterococcus faecalis*, while no activity was observed for the Gram-negative ones [142]. Identical results were observed for an acetone extract from *A. nodosum*, which also produced more effective inhibition towards Gram-positive (MIC of 0.25 and 0.2 mg/mL for *M. luteus* and *S. aureus*, respectively) than Gram-negative (MIC of 0.4 and 0.5 mg/mL for *E. coli* and *Enterococcus aerogenes*, respectively) bacteria. Notably, the inhibitory effectiveness of this extract towards Gram-positive and Gram-negative bacteria was respectively 25–30- and 12–15-times stronger than those of ethylparaben, sodium benzoate and potassium sorbate, which are three important bactericides used as food preservatives [143]. On the other hand, Wang et al. [144] observed that a purified phlorotannin extract of *A. nodosum* origin exhibited strong bactericidal activity against *E. coli* O157:H7, and a complete eradication of this microorganism was observed after 6 h treatment with extract at 50 μg/mL. Furthermore, in combination with a silver-zeolite, *A. nodosum* aqueous phenolic-rich extract obtained by alginate precipitation and a series of filtrations resulted in a complete inhibition of the film formation of *Streptococcus gordonii* alone and altered the film formation of co-cultured *Porphyromonas gingivalis* and *S. gordonii*, thus indicating a possible therapeutic approach for preventing and/or treating periodontal diseases [126]. Fungicidal properties were described for *F. spiralis* phlorotannin-purified acetone 70% extract, which exhibited particular inhibitory effects against the growth of several dermatophytes, revealing MICs of 3.9–31.3 mg/mL DW for *Trichophyton rubrum* > *Epidermophyton floccosum* > *Trichophyton mentagrophytes* = *Microsporum canis* > *Microsporum gypseum*, thus being of some interest for the development of skincare products for the treatment of dermatophytosis [145]*.* In addition, *F. vesiculosus* was shown to be a rich source of polysaccharides and polyphenols with the capacity to inhibit both HIV-induced syncytium formation and reverse transcriptase activity [146].

Photoprotective activity is another biological property that has been described for phenolics from Fucaceae as well. The phenolic-rich water-soluble fraction from *F. vesiculosus* and *A. nodosum* acetone 70% extracts revealed moderate photoprotective effects in vivo, as they could prevent the UV-B-exposed zebrafish embryos from dying, although a big percentage of these embryos presented low-level malformations. Notwithstanding, the number of normal embryos was higher in the presence 0.4 mg PE/mL of *F. vesiculosus* extract (17%) than in the presence of the same concentration of *A. nodosum* (8.3%), which is very likely due to the differences in the phenolic profile between species and consequently their different radical scavenging activities [147].

Consumption of Fucaceae seaweeds may also have a significant impact on the control of hypertensive conditions. In this context, a methanolic extract from *F. spiralis* was reported for its capacity to inhibit angiotensin I-converting enzyme, a key player in the control of blood pressure, by approximately 80%. In addition, the fractionation of this extract according to their molecular weight (<1 kDa, 1–3 kDa and >3 kDa) resulted in distinct inhibition abilities towards the enzyme, being the strongest (almost 90% inhibition) observed for the >3 kDa fraction at 200 μg/mL, which was almost as effective as captopril (i.e., a pharmaceutical anti-hypertensive agent) that caused 97% of inhibition for the same concentration [148]. *S. siliquosa* ethanol 95% extract has shown good inhibitory effects towards this enzyme as well (45% at 164 μg/mL), although this was considerably less effective than captopril (33% inhibition at 1.6 ng/mL) [149].

Recently, Kellogg et al. [131] observed that the treatment of 3T3-L1 adipocytes with 100 μg/mL of an ethyl acetate fraction that was obtained from a *F. distichus* crude methanolic extract resulted in a reduction of cellular lipid accumulation to 77.5%. Moreover, the authors further observed that, at 50 μg/mL, a fucophlorethol-rich subfraction obtained by an addition purifying step onto a Sephadex LH-20 column not only reduced lipid accumulation in the same cellular model down to 45.9%, but also had the capacity to inhibit leptin mRNA expression close to 0% and enhance that of adiponectin in 20% compared to the untreated control. These results were even more pronounced than those obtained for dexamethasone, which only caused a 20% reduction of the leptin and negatively interfered with the adiponectin expression, reducing it by approximately 50%. Therefore, these data suggest that *F. distichus* phlorotannins may exert anti-obesity effects through regulation of lipid metabolism.

It should be noted that phlorotannins are endowed with several other biological properties such as neuroprotective, cardioprotective, antiallergic, anti-arthritis and many others. However, it must also be emphasized that these properties are very frequently reported for phlorotannins from brown algae species such as *Ecklonia* spp., *Eisenia* spp. and *Ishige okamurae*, which are abundant in phlorotannins (such as eckol, dieckol and several derivatives) that have different structural features when compared to the typical phlorotannins found in Fucaceae [52,150,151]. Hence, further studies are necessary in order to clarify the possible targeting of Fucaceae typical phlorotannins in such mechanisms.

## 4. Bioavailability

Dietary habits are the major source of polyphenols. However, the biological activity of these compounds in vivo is critically influenced by their bioaccessibility, absorption and metabolism [152]. Since very few data concerning the bioavailability of phlorotannins are currently available, it is common to consider that this group of compounds follow an identical behavior to that of plant polyphenols, which are better absorbed in the large intestine after undergoing an extensive transformation by enzymatic activity or colon microbial fermentation [153,154].

Until recently, the bioavailability of phlorotannins was still an unexplored subject. However, the earliest studies in this field are already emerging. A preliminary approach was carried out by Bangoura et al. [155,156], who observed that the phlorotannin concentration in the flesh of abalones was raised after feeding them either with *Ecklonia cava* or *Ecklonia stolonifera*, i.e., two brown algae species rich in these compounds.

More recently, Corona and co-workers [85] conducted a study aiming to determine the gastrointestinal stability and bioavailability of a food-grade phlorotannin extract from *A. nodosum*. In more detail, this extract was submitted to an in vitro gastric and ileal digestion followed by colonic bacteria fermentation and, ultimately, a dialysis filtration to simulate the absorption into the circulation. Through HPLC-MS, the authors could identify 11 compounds in the dialysate, four of them corresponding to hydroxytrifuhalol A, a C–O–C dimer of phloroglucinol, diphlorethol/difucol and 7-hydroxyeckol, which had been previously identified in the crude extract of *A. nodosum*, and seven new uncharacterized compounds that corresponded to in vitro-absorbed metabolites. Some of these compounds were further detected on urine samples of human volunteers who were administered with a single capsule containing 100 mg of the *A. nodosum* extract, thus confirming that they were absorbed into the blood circulation in vivo. In plasma, the total level of phlorotannins/metabolites detected varied between 0.011 and 7.757 µg/mL, while in urine, the values ranged between 0.15–33.52 µg/mL, and although some metabolites were found in samples collected at 2–4 h after capsule ingestion, the majority were detected at late time points, indicating that the high molecular weight phlorotannins were poorly absorbed in the upper tract and went through colonic fermentation, which resulted in the formation of lower molecular weight derivatives that were more likely to be absorbed. During the passage through the digestive tract, phenolic compounds are known to undergo extensive modification by glucosidase enzymes, phase I enzymes, including cytochrome P450, and phase II enzymes (glucuronosyltransferases, sulfotransferases) found both in the small intestine and the liver [157]. Indeed, in this work, the authors found that some metabolites were only detectable in blood or urine samples after an enzymatic treatment with glucuronidase or sulfatase, while others were only observable in untreated samples, indicating that these compounds corresponded to the conjugated metabolites. On the other hand, some compounds were detected in samples either with or without enzymatic treatment, which means that these were the unconjugated metabolites [85].

Based on these data, it seems that, similarly to what happens to plant polyphenols, phlorotannins may undergo different modifications during their transit in the gastrointestinal tract, and the resultant metabolites might represent active forms that will pass through the gut barrier and exert their physiological and biological functions in the organism [158,159].

## 5. Concluding Remarks

In conclusion, Fucaceae seaweeds are a valuable source of phlorotannins, which have drawn much attention during recent years due to their numerous possible therapeutic properties. Common features of phlorotannin extracts from Fucaceae include antioxidant effects through scavenging of ROS or enhancement of intracellular antioxidant defenses, antidiabetic properties through their acarbose-like activity and capacity to increase adipocytes glucose uptake and β-pancreatic cells resistance to high-glucose oxidative stress, anti-inflammatory effects through inhibition of several pro-inflammatory mediators and antitumor properties through activation of apoptosis on cancerous cells and inhibition of metastasis. Other important biological activities have been demonstrated, such as antimicrobial, anti-hypertensive, anti-obesity and photoprotective activities. Besides, the bioavailability of phlorotannins is presently suggested to resemble that of plant tannins, with the majority of these compounds being modified by the gut microflora and the resultant metabolites possibly representing true bioactive forms. In sum, it can be suggested that Fucaceae phlorotannins present powerful and versatile bioactivities that grant them great potential for exploitation as renewable feedstocks for the development of new nutraceutical, cosmetic and pharmaceutical products.

## Figures and Tables

**Table 1:** Selected studies of antioxidant activity of phlorotannin extracts of some Fucaceae, as measured by in vitro and in vivo biological models.

Extraction Method	Model	Treatment Conditions	Effect	References
*F. vesiculosus*				
EtOH 80% → fractionation with *n*-Hex and EtOAc → subfractionation of EtOAc in Sephadex LH-20	PMA-treated mononuclear cells from human blood	10 μM PMA + 1.5 μg/mL of 6 different EtOAc sub-fractions	All sub-fractions (except the 4th) ↓ ROS levels below 65%	[91]
MeOH 60%	Caco-2 cells	100 μg/mL of extract for 24 h	↑ GSH levels by 31.9%	[88]
MeOH 60%	H_2_O_2_-induced Caco-2 cells	24 h pre-treatment with 100 μg/mL of extract for 24 h + 200 μM H_2_O_2_	Restored SOD levels from 64.9 to 89% and ↓ 9.5% of the DNA damage	[39,88]
MeOH 60%	*t*-BHP-induced Caco-2 cells	100 μg/mL of extract for 24 h + 200 μM *t*-BHP	↓ apx. 12% DNA damage in *t*-BHP-induced cells	[98]
Ext1: EtOH 35% Ext2: EtOH 70%	In vitro: PMA or LPS-induced Raw 264.7 cells In vivo: Sprague–Dawley rats	In vitro: 100 ng/mL PMA or LPS + different concentrations of extracts In vivo: oral treatment with 200 mg/kg/day during 4 weeks	In vitro: Ext2: ↓ of O_2_^●−^ in PMA-induced cells (IC_50_ = 31 μg/mL), Ext1: ↓ of O_2_^●−^ in both cell models (IC_50_ = 38 and 39 μg/mL, respectively); In vivo: Ext2: ↑ reducing power, PON-1 activity and O_2_^•−^ scavenging activity in the blood plasma (29%, 33% and 25%, respectively)	[97]
*F. serratus*				
MeOH 60%	Caco-2 cells	100 μg/mL of extract for 24 h	↑ GSH levels by 37.4%	[88]
MeOH 60%	*t*-BHP or H_2_O_2_-induced Caco-2 cells	100 μg/mL of extract for 24 h + 1 mM *t*-BHP or 200 μM H_2_O_2_	Restored SOD levels in both *t*-BHP and H_2_O_2_-induced cells from 73.9–108% and 64.9–89.5%, respectively, and ↓ 13.2% of the H_2_O_2_-induced DNA damage	[39,88]
Ext1: H_2_O Ext2: EtOH 80%	*t*-BHP-induced Caco-2 cells	100 μg/mL of extracts for 24 h + 1 mM *t*-BHP	Both extracts ↓ apx. 13% DNA damage in *t*-BHP-induced cells	[98]
*A. nodosum*				
Extract with 18% phlorotannins	*t*-BHP-induced ARPE-19 and WKD cells	0.1–0.5% extract for 20 min + 500 μM *t*-BHP	↓ ROS production close to the negative control on cells treated with 0.2% extract	[38]
MeOH 60% → digestion with pepsin at 37 °C and pH 2 → digestion with pancreatin/bile extract at 37 °C pH 6.9 → dialysis with cutoff at 1 kDa	*t*-BHP-induced HepG-2 cells	0.5–50 μg/mL of extract for 20 h + 400 μM *t*-BHP	↓ ROS and lipid, restored GSH levels to apx. 75% and regulated the activity of GSH-px, GSH-red GSH-tr	[99]
MeOH 60%	Caco-2 cells	100 μg/mL of extract for 24 h	↑ GSH levels by 35.5%	[88]
MeOH 60%	H_2_O_2_-induced Caco-2 cells	100 μg/mL of extract for 24 h + 200 μM H_2_O_2_	Restored SOD levels from 64.9–89.5%	[88]
Ext1: H_2_O Ext2: EtOH 60% Ext3: EtOH 80%	*t*-BHP or H_2_O_2_-induced Caco-2 cells	100 μg/mL of extracts for 24 h + 1 mM *t*-BHP or 200 μM H_2_O_2_	Ext1: ↓ 20% H_2_O_2_-induced DNA damage; Ext2: ↓ apx. 15% *t*-BHP -induced DNA damage, Ext3: ↓ apx. 13% DNA damage in both models	[98]
*P. canaliculata*				
MeOH 60%	Caco-2 cells	100 μg/mL of extract for 24 h	↑ GSH levels by 38.7%	[88]
MeOH 60%	*t*-BHP or H_2_O_2_-induced Caco-2 cells	100 μg/mL of extract for 24 h + 1 mM *t*-BHP or 200 μM H_2_O_2_	Restored SOD levels from 73.9–97% and 64.9–97.4%, respectively	[39,88]

apx., approximately; EtOAc, ethyl acetate; EtOH, ethanol; Ext, extraction; GSH, glutathione; GSH-px, glutathione peroxidase; GSH-red, glutathione reductase; GSH-tr, glutathione transferase; LPS, lipopolysaccharide; MeOH, methanol; *n*-Hex, *n*-hexane; PON-1, paraoxonase 1; SOD, superoxide dismutase; PMA, phorbol-12-myristate-13-acetate; ROS, reactive oxygen species; *t*-BHP, and *tert*-butyl hydroperoxide. Cell lines: ARPE-19, human retinal pigment epithelium; Caco-2, human epithelial colorectal adenocarcinoma; HepG-2, liver hepatocellular carcinoma; Raw 264.7, murine macrophages; and WKD, human conjunctival cells.

**Table 2:** Selected studies of the anti-diabetic activity of phlorotannin extracts of some Fucaceae, as measured in vitro and in vivo.

Extraction Method	Model	Test Conditions	Effect	References
***F. vesiculosus***				
Sequential extraction with CHCl_3_ → EtOH 96% → Ac 70%	Measurement of α-glucosidase activity	Crescent concentrations of extracts	EtOH and Ac extracts had the highest inhibitory activity (IC_50_ = 4.4 and 0.34 μg/mL, respectively)	[111]
Ext1: H_2_O Ext2: EtOH	Measurement of α-glucosidase and α-amylase activities	0.1–1000 μg/mL of extracts	↓ enzymatic activity (α-glucosidase: IC_50_ = 0.32 and 0.49 μg/mL, respectively; α-amylase: IC_50_ = 59.1 and 63.5 μg/mL, respectively)	[47]
Ac 70% → fractionation with DCM, EtOAc and But → subfractionation of EtOAc in Sephadex LH-20 (F1–F4)	BSA-methylglyoxal and BSA-glucose assay	Crescent concentrations of fractions or sub-fractions	Strong ↓ BSA glycation by subfractions, (EC_50_ apx. 0.16 mg/mL for F1–F4 in BSA-methylglyoxal and 0.05 mg/mL for F1 and F2 in BSA-glucose)	[116]
***F. distichus***				
EtOH 80% → Fractionation with *n*-hex, EtOAc, 1-But → subfractionation of EtOAc in Sephadex LH-20	Measurement of α-glucosidase and α-amylase activities	1.5–200 μg/mL of subfractions	Subfraction 22 showed ↑ inhibitory activity (IC_50_ = 0.89 and 13.98 μg/mL, respectively)	[115]
***A. nodosum***				
EtOH 50% at 80 °C → Fractionation with EtOAc and 1-But → purification in C18 column	Measurement of α-glucosidase activity	Crescent concentrations of fractions	Purified fraction showed ↑ inhibitory activity (IC_50_ = 24 μg/mL)	[117]
Sequential extraction with CHCl_3_ → EtOH 96% → Ac 70%	Measurement of α-glucosidase activity	Crescent concentrations of extracts	Ac extracts showed ↑ inhibitory activity (IC_50_ = 0.72 μg/mL)	[111]
H_2_O at 80 °C from algae collected at different seasons	Measurement of α-glucosidase activity	0.05–0.5 μg/mL of extract	Summer extracts have ↑ inhibitory activity (IC_70_ = 2.23 μg/mL)	[113]
Ext1: H_2_O Ext2: EtOH	Measurement of α-glucosidase and α-amylase activities	0.1–1000 μg/mL of extracts	↓ enzymatic activity (α-glucosidase: IC_50_ = n.d.; α-amylase: IC_50_ = 44.7 and 53.6 μg/mL, respectively)	[47]
EtOH 50%	2-deoxyglucose-cultured 3T3-L1 cells	50–400 μg/mL of extract for 20 min + 1 μCi/mL 2-deoxyglucose	↑ basal glucose uptake by 3-fold at 400 μg/mL	[117]
ACN:0.2% CH_2_O_2_ (1:1) → purification in SPE column → fractionation in Sephadex LH-20	Measurement of α-glucosidase and α-amylase activities in absence or presence of acarbose	Phlorotannin fraction: 2.5–100 μg GAE/mL for α-glucosidase and 50–400 μg GAE/mL for α-amylase; acarbose + phlorotannin fraction: 1 μg/mL + 0.1 μg/GAE –0.25 μg/mL + 0.025 μg/GAE	↓ enzymatic activity (α-glucosidase: IC_50_ = 10 μg GAE/mL; α-amylase: IC_50_ = 0.15 μg GAE/mL). ↓ acarbose concentration needed for an effective enzymatic inhibition (from 1–0.5 μg/mL)	[118]
***P. canaliculata***				
MeOH 70%	In vitro: measurement of sucrase and maltase activities In vivo: sucrose-fed Wistar rats	In vitro: 0–16.7 mg/mL extract In vivo: oral administration of 1 mg/kg of extract + 0.5 mg/kg of sucrose	In vitro: ↓ enzymatic activity (IC_50_ = 2.24 and 2.84 mg/mL, respectively) In vivo: ↓ postprandial blood glucose levels	[112]
***A. nodosum* combined with *F. vesiculosus***				
Commercial hot water extract InSea2^TM^ (10% polyphenol content in CAE)	In vitro: measurement of α-glucosidase and α-amylase activities In vivo: Wistar rats fed with corn starch + safflower oil	In vitro: 1.25–25 μg/mL of InSea2^TM^ In vivo: oral administration of 7.5 mg/kg of InSea2^TM^ + 2 mL/kg of starch and oil (1:1)	In vitro: ↓ enzymatic activity (IC_50_ = 2.8 and 5 μg/mL, respectively) In vivo: ↓ 90% postprandial blood glucose and ↓ 40% insulin peak	[119]
Commercial hot water extract InSea2^TM^ (10% polyphenol content in CAE)	Human trial	Oral administration of two capsules (500 mg) 30 min prior to carbohydrate ingestion	↓ insulin incremental area of the curve by 12.1% and ↑ insulin sensitivity by 7.9%	[120]

Ac, acetone; apx., approximately; BSA, bovine albumin serum; But, butanol; CAE, chlorogenic acid equivalents; DCM, dichloromethane; EtOAc, ethyl acetate; EtOH, ethanol; Ext, extraction; HCl, chloridric acid; GAE, gallic acid equivalents; SPE column, solid-phase extraction column; and ROS, reactive oxygen species; Cell lines: INS-1, rat pancreatic β-cells; and 3T3-L1, preadipocytes.

**Table 3:** Selected studies of the anti-inflammatory activity of phlorotannin extracts of some Fucaceae, as measured in in vitro and ex vivo biological models.

Extraction method	Model	Test Conditions	Effect	References
***F. vesiculosus***				
H_2_O	LPS-induced porcine colonic tissue ex vivo	1 mg/mL extract + 10 µg/mL LPS	↓ expression of the genes *IL17A*, *IL8*, *CCL2*, *CXCL2*, *CXCL10*, *CXCL11*, *ICAM1*, *VCAM1*, *TLR4*, *TLR7*, *NFKB1*, *RELB*, *MAP3K8*, *CJUN*, *PTGS2*, *C5* and *LYZ* >2× compared to the control	[128]
EtOH 35%	PMA-stimulated RAW 264.7	100 ng/mL PMA + different concentrations of extracts	↓ production of NO^●^ (IC_50_ = 37 µg/mL)	[97]
***F. serratus***				
H_2_O	LPS-induced porcine colonic tissue ex vivo	1 mg/mL extract + 10 µg/mL LPS	↓ expression of the genes *IL8*, *IL6* and *TNFA* below 0.70, 0.69 and 1.15× compared to LPS control, respectively	[42]
***F. distichus***				
MeOH 80% → fractionation with *n*-hex, EtOAc and 1-But → subfractionation of EtOAc in flash chromatography	LPS-induced RAW 264.7 cells	12.5–50 µg/mL a subfraction rich in fucophlorethols for 1 h + 1 µg/mL LPS	↓ expression of IL-1β, IL-6, IL-17, TNF-α, MCP-1, iNOS, COX-2, ICAM-1, TLR-4 and TLR-9 in a dose-dependent manner	[131]
***A. nodosum***				
Extract with 18% phlorotannins	LPS-induced U937 cells	0.05–0.2% of extract for 2 h + 0.5 µg/mL LPS	↓ levels of TNF-α and IL-6 close to control	[38]
H_2_O → alginate precipitation *→* ultrafiltration	LPS-induced U937 cells	0.1 µg extract for 2 h + 0.5 µg/mL LPS	↓ levels of TNF-α by 94% and IL-6 by 84%	[126]
EtOH 80%	LPS-induced porcine colonic tissue ex vivo	1 mg/mL extract + 10 µg/mL LPS	↓ expression of the genes *IL8*, *IL6* and *TNFA* below 0.99, 0.75 and 1.01× compared to LPS control, respectively	[42]
EtOH 80%	TNF-α-induced Caco-2 cells	0.1–1 mg/mL extract + 10 ng/mL TNF-α	↓ expression of the genes *IL8*, *TNFA*, *IL1B*, *IL18*, *CSF1*, *CXCL10*, *CCL5*, *NFKB2*, *IKBKB*, *PTGS2* and *MIF* by >2×	[127]
EtOH 80% *→* dialysis fractionation into three *M*_w_ fractions (<3.5 kDa, 3.5–100 kDa, >100 kDa)	LPS-induced porcine colonic tissue ex vivo	1 mg/mL extract or *M*_w_ fractions + 10 µg/mL LPS	↓ expression of the genes *LYZ*, *IL8*, *PTGS2*, *TLR6*, *CXCL10*, *IL6*, *CXCL11*, *ICAM*, *NFKB1* and *CXCL2* by >2× either by the crude extract or the three *M*_w_ fractions	[127]

COX-2, cyclooxygenase-2; EtOAc, ethyl acetate; IL, interleukin; iNOS, inducible nitric oxide synthase; LPS, lipopolysaccharide; *M*_w_, molecular weight; NO^●^, nitric oxide; PMA, phorbol-12-myristate-13-acetate; and TNF-α, tumor necrosis factor-α. Cell lines: RAW 264.7, murine macrophages; U937, human leukemic monocytes; and Caco-2, human colon epithelium.

**Table 4:** Selected studies of the antitumor activity of phlorotannin extracts of some Fucaceae, as measured in in vitro biological models.

Extraction Method	Model	Test Conditions	Effect	References
***F. vesiculosus***				
Acetone 99.5% → purification by HPLC	PancTu1, Panc89, Panc1 and Colo357 cells	12.5–100 µg/mL of purified extract	↓ cell proliferation, ↑ cell cycle inhibitors (IC_50_ = 17.35 µg/mL, 17.5 µg/mL, 19.23 µg/mL and 28.9 µg/mL, for each cell line, respectively)	[133]
H_2_O → precipitation → normal phase HPLC → reversed phase HPLC→ F15 + F16	Panc89 and PancTu1 cells	0.2–200 µg/mL of fractions	↓ cell proliferation (F15: IC_50_ = 15.2 and 18.3 μg/mL, respectively; F16: IC_50_ = 16.4 and 16.2 μg/mL)	[134]
***F. spiralis***				
Ext1: DCM Ext2: MeOH 100% Ext3: *n*-hex fraction of Ext2	HeLa cells	Crescent concentrations of dichloromethane extract	↑ apoptosis, with Ext1 showing highest activity (IC_50_ = 10.7 μg/mL)	[135]
Ac 70% → purification with cellulose	Hyaluronidase activity measurement	0.5–2.25 mg/mL of extract	↓ enzymatic activity (IC_50_ = 0.73 mg/mL dry weight)	[136]
***A. nodosum***				
ACN:0.2% CH_2_O_2_ (1:1) → purification in SPE columns	Caco-2 cells	15–42.5 µg/mL of extract	↓ cell proliferation (IC_50_ = 33 μg/mL)	[114]

Ac, acetone; ACN, acetonitrile; DCM, dichloromethane; Ext, extraction; F15, fraction 15; F16, fraction 16; HPLC, high performance liquid chromatography; MeOH, methanol; SPE column, and solid-phase extraction column. Cell lines: Caco-2, human colon cancer; HeLa, human cervix carcinoma; PancTu1, human pancreatic cancer; Colo357, human pancreatic adenosquamous carcinoma; Panc89, human pancreatic cancer; and Panc1, pancreatic carcinoma.

## Abbreviations

AGEs	Advanced Glycated End-Products
BSA	Bovine Serum Albumin
CAT	Catalase
COX-2	Cyclooxygenase-2
DP	Degree of Polymerization
DPPH^●^	1,1-Diphenyl-2-Picrylhydrazyl Radical
FRAP	Ferric Reducing Antioxidant Power
GAE	Gallic Acid Equivalents
GSH	Glutathione
GSH-px	Glutathione Peroxidase
GSH-red	Glutathione Reductase
GSH-tr	Glutathione Transferase
HPLC	High Performance Liquid Chromatography
IL	Interleukin
iNOS	Inducible Nitric Oxide Synthase
LPS	Lipopolysaccharide
MIC	Minimum Inhibitory Concentration
MS	Mass Spectrometry
NF-κB	Nuclear Factor-κB
NMR	Nuclear Magnetic Spectroscopy
Nrf2	Nuclear Factor (Erythroid-Derived 2)-Like 2
ORAC	Oxygen Radical Absorbance Capacity
PE	Phloroglucinol Equivalents
PMA	Phorbol-12-Myristate-13-Acetate
PON-1	Paraoxonase-1
RNS	Reactive Nitrogen Species
ROS	Reactive Oxygen Species
SOD	Superoxide Dismutase
SPE	Solid Phase Extraction
*t*-BHP	*tert*-Butyl Hydroperoxide
TBARS	Thiobarbituric Acid Reactive Substances
TE	Trolox Equivalents
TLR	Toll-Like Receptor
TNF-α	Tumor Necrosis Factor-α
TPC	Total Phenolic Content
UV	Ultraviolet
UPLC	Ultra-Performance Liquid Chromatography
